# Assessment of genetic variations among highly endangered medicinal plant *Bacopa monnieri* (L.) from Central India using RAPD and ISSR analysis

**DOI:** 10.1007/s13205-012-0059-3

**Published:** 2012-04-07

**Authors:** Niraj Tripathi, Devendra Singh Chouhan, Navinder Saini, Sharad Tiwari

**Affiliations:** 1 Biotechnology Centre, Jawaharlal Nehru Agricultural University, Jabalpur, 482004 India; 2 Vivekananda Hill Agricultural Research Institute, Almora, 263601 India

**Keywords:** *Bacopa monnieri*, Genetic variation, Inter simple sequence repeats (ISSR), Polymorphism, Random amplified polymorphic DNA (RAPD)

## Abstract

Genetic variations of 15 Brahmi (*Bacopa monnieri* L.) accessions were evaluated using random amplified polymorphic DNA (RAPD) and inter simple sequence repeats (ISSR) markers. During RAPD analysis, amplification of genomic DNA of the 15 accessions by 22 primers generated 197 fragments, of which 187 were polymorphic with an average of 8.95 bands per primer. The amplified products varied in size from 2,200 to 250 bp. Twenty-five selected ISSR primers produced 284 bands across 15 accessions, of which 270 were polymorphic with an average of 10.80 bands per primer. The PIC value ranges from 0.363 to 0.908 for RAPD primers, while 0.419 to 0.836 in case of ISSR. The size of amplified bands ranged from 2,800 to 240 bp. Similarity index values ranged from 0.16 to 0.95 (RAPD), 0.18 to 0.98 (ISSR) and 0.179 to 0.945 for pooled ISSR and RAPD markers data. Mantel test revealed the similar distribution pattern of the polymorphism between RAPD and ISSR markers and the correlation co-efficient (*r*) was 0.71384. The results indicated that both of the marker systems RAPD and ISSR, individually or combined can be effectively used in determination of genetic relationship among *B. Monnieri* accessions collected from different parts of Central India. It could be concluded that the information of genetic similarities and diversity among Brahmi accessions is necessary for their conservation and breeding programs.

## Introduction

*Bacopa monnieri* (L.) Penn. commonly known as ‘Neera-Brahmi’ of family Scrophulariaceae is a small prostrate herb that grows wild in marshy and damp places near water logs throughout India. *B. monnieri* (Brahmi) has been used for centuries as a traditional Ayurvedic medicine to enhance memory and to prepare popular ayurvedic preparations like ‘Brahmirasayanam’ and ‘Brahmighritam’ (Govindarajan et al. 2005; Prasad et al. 2008). Bacoside A and B are the major active compounds of Brahmi. Medicinal and aromatic plant utilizations and conservation have attracted global attention due to their over exploitation (Chomachalow 1980; Parrotta 2001). According to a report of National Medicinal Plants Board (NMPB) and Technology Information Forecasting and Assessment Council (TIFAC) Department of Science and Technology, Government of India, (2007), the annual market demand for brahmi during 2000 was around 1,000 tones which increased many fold due to its potential uses in ayurvedic system of medicine to treat variety of ailments. The rapidly rising requirement has placed *B. monnieri* as the second most priority species among the most important Indian medicinal plants (Rajani 2008) and denoted among 32 medicinal plants identified for cultivation and conservation by the National Medicinal Plants Board, Government of India (National Medicinal Plants Board 2004). Recently, *B. monnieri* has been identified among the seven important medicinal plants recommended for immediate attention and included in the list of highly endangered medicinal plants of India by NMPB and Technology Information Forecasting and Assessment Council (TIFAC), Department of Science and Technology, Government of India (http://www.nmpb.nic.in/prioritisemedicinalplants.htm). The continuous exploitation of *B. monnieri* from the natural habitat has now resulted in the depletion of natural population. The present status of the plant attracted attention of biotechnologists to conserve this important medicinal herb. Morphological traits display continuous phenotypic range and work as quantitative traits dominated by multigenes (Chen et al. 1990). In addition, morphological characteristics are considerably affected by the environmental factors. Therefore, accurate identification based merely on morphological traits becomes difficult and reinforce the importance of molecular markers for precise identification.

The molecular approach for the identification of plant varieties/genotypes seems to be more effective as compared to traditional morphological markers as it directly access the hereditary information and makes it possible to understand the relationships between individuals (Paterson et al*.*^1991^). PCR-based molecular markers are widely used in many plant species for identification, phylogenetic analyses, population studies and genetic linkage mapping (Williams et al. 1990). Both RAPD and ISSR markers have proved to be a reliable, easy to generate, inexpensive and versatile set of markers that rely on repeatable amplification of DNA sequence using single primer. The RAPD and ISSR markers have been used to study the genetic variability among species or natural populations and in the identification of genotypes (Wilde et al*.*^1992^; Koller et al. 1993; Lashermes et al. 1993; Wilkie et al. 1993; Wolff and Peters-Van Run 1993; Pharmawati et al*.*^2004^; Mohapatra and Rout 2005; Barik et al. 2006). The genetic diversity analysis of Indian *B. monnieri* accessions using molecular markers is quite scanty. Only two reports are available on the use of RAPD marker for *B. monnieri* (Darokar et al. 2001; Karthikeyan et al. 2011) on the other hand, there is no evidence available for the use of ISSR markers with this species. The ISSR analysis is a very useful molecular tool for studying population genetics on a wide range of plant species, as well as for identifying species, cultivars, or population of the same species (Zietkiewicz et al. 1994; Raina et al. 2001; Wang et al. 2009). In this first report with two PCR-based DNA (RAPD and ISSR) markers, we describe the feasibility for the identification of phylogenetic relationship among *B. monnieri* accessions collected from Central India.

## Materials and methods

### Plant materials

A total of 15 accessions (Table 1) of *B monnieri* (5 plants of each accession) were collected from various locations of Central India and maintained at Biotechnology Centre, Jawaharlal Nehru Agriculture University, Jabalpur, India.

**Table 1:** Location of germplasm collection and morphological characteristics of 15 accessions of *Bacopa monnieri*

Sl.	Place of origin	Geographical location		Accessions label	Stem	Leaf size^a^	Leaf color	Plant type
		Latitude (N)	Longitude (E)					
1	Hoshangabad	22°46′	77°45′	BM-1	Thin green	Large	Yellow-green	Semi-erect
2	Dewas	22°58′	78°06′	BM-2	Thin green	Large	Yellow-green	Semi-erect
3	Khandwa	21°82′	76°34′	BM-3	Thick green	Small	Green	Spreading
4	Indore	22°44′	75°50′	BM-4	Thin green	Small	Green	Spreading
5	Bhopal	23°16′	77°36′	BM-5	Thick green	Small	Green	Spreading
6	Seoni	22°06′	79°35′	BM-6	Thick green	Small	Green	Semi-erect
7	Rewa	24°32′	81°18′	BM-7	Thick green	Small	Green	Spreading
8	Badwani	22°03′	74°57′	BM-8	Thin green	Small	Green	Spreading
9	Amarkantak	22°67′	81°75′	BM-9	Thin green	Small	Green	Spreading
10	Shivpuri	25°4′	77°44′	BM-10	Thin green	Small	Green	Semi-erect
11	Satna	24°34′	80°55′	BM-11	Thin green	Medium	Dark green	Spreading
12	Singrauli	24°12′	82°39′	BM-12	Thick green	Small	Green	Spreading
13	TFRI, Jabalpur	23°10′	79°59′	BM-13	Thick green	Small	Green	Spreading
14	JNKVV, Jabalpur	23°10′	79°59′	BM-14	Thin green	Medium	Dark green	Spreading
15	Indore	22°44′	75°50′	BM-15	Thick green	Small	Green	Spreading

^a^Large (>70 mm^2^), medium (60–70 mm^2^), small (50–60 mm^2^)

### DNA extraction

Total genomic DNA was extracted using a modified CTAB method based on the protocol of Doyle and Doyle (1990). Young leaves were used for DNA extraction from each plant of every accession. Quality of DNA was tested by submerged horizontal agarose gel (0.8 %) electrophoresis and visualized with UV light.

### PCR analysis

PCR amplifications were performed in a programmable thermocycler (Thermo Hybaid®). Each sample was amplified in a reaction mixture containing 50 ng genomic DNA, *Taq* polymerase 1 unit (Sigma Co., USA), 10× PCR buffer with 2.5 mM MgCl_2_ and 200 μM of each dNTP mixture (Sigma Co., USA), 15 pmol of 10-mer RAPD and ISSR primers (Operon Technologies, USA; Table 2). Cycling parameters for ISSR were adjusted to 5 min at 94 °C for pre-denaturation, 39 cycles each of 1 min at 94 °C for denaturation, 1 min for annealing at 45/50/55 °C, 2 min at 72 °C for extension and a final extension at 72 °C for 5 min. For RAPD marker analysis, the PCR reaction mix and program profile were similar to ISSR markers analysis except the annealing temperature which was adjusted to 37 °C. After cooling to 4 °C, amplified products were stored at −20 °C until electrophoresis. Amplified products were separated on 1.5 % agarose gel (Sigma, USA) in 1× TAE buffer with 1 Kb plus ladder (Fermentas, USA) to determine the size of amplified DNA fragments. Gels were run for 4 h at 65 V, stained with ethidium bromide and documented with gel documentation system (Syngene, UK).

**Table 2:** Data for 22 RAPD and 25 ISSR primers used for analysing 15 accessions of *Bacopa monnieri*

Code	Total bands	Polymorphic bands	% Polymorphism	PIC
*RAPD*				
OPB-08	10	10	100.00	0.783
OPB-10	8	8	100.00	0.701
OPB-18	8	8	100.00	0.646
OPC-05	11	11	100.00	0.731
OPA-11	9	9	100.00	0.908
OPC-06	9	9	100.00	0.652
OPC-09	7	7	100.00	0.668
OPAA-02	5	4	80.00	0.557
OPAA-04	11	9	81.81	0.570
OPAB-07	11	11	100.00	0.724
OPAB-08	9	8	88.88	0.602
OPAC-12	10	10	100.00	0.593
OPAI-03	10	7	70.00	0.363
OPAI-05	8	6	75.00	0.459
OPAC-13	8	8	100.00	0.516
OPAC-14	7	7	100.00	0.506
OPAI-06	11	11	100.00	0.691
OPBB-04	7	6	85.71	0.587
OPBB-07	10	10	100.00	0.778
OPAD-06	7	7	100.00	0.738
OPAH-05	11	11	100.00	0.573
OPBB-08	10	10	100.00	0.817
Total	197	187	–	
Average	8.95	8.50	94.6	0.644
*ISSR*				
ISSR 807	13	13	100	0.706
ISSR 808	10	8	80	0.572
ISSR 810	15	15	100	0.730
ISSR 811	16	16	100	0.661
ISSR 812	6	6	100	0.459
ISSR 814	10	10	100	0.731
ISSR 824	9	9	100	0.731
ISSR 825	10	10	100	0.673
ISSR 827	11	11	100	0.638
ISSR 834	14	14	100	0.641
ISSR 815	11	11	100	0.591
ISSR 822	11	11	100	0.749
ISSR 823	9	9	100	0.837
ISSR 836	13	11	84.61	0.464
ISSR 840	12	12	100	0.699
ISSR 842	16	15	93.75	0.711
ISSR 844	12	11	91.66	0.697
ISSR 859	10	10	100	0.704
ISSR 876	15	13	86.66	0.651
ISSR 881	12	12	100.00	0.733
ISSR 885	10	8	80.00	0.610
ISSR 889	13	12	84.61	0.754
ISSR 890	8	7	87.50	0.761
ISSR 891	12	11	91.66	0.686
ISSR 895	6	6	100.00	0.419
Total	284	270	–	
Average	11.36	10.8	95.22	0.664

### Data analysis

Evaluation of fragment patterns was carried out by similarity index. Reproducible bands were scored manually as ‘1’ or ‘0’ for presence or absence of the bands. Polymorphic information content (PIC) values were calculated for each RAPD and ISSR primer according to the formula: PIC = 1 − Σ(*P*_*ij*_)^2^, where *P*_*ij*_ is the frequency of the *i*th pattern revealed by the *j*th primer summed across all patterns revealed by the primers (Botstein et al. 1980). The final RAPD and ISSR data generated were used to calculate pairwise similarity co-efficients (Jaccard 1908) using the similarity for qualitative data (SIMQUAL) format of NTSYS-pc version 2.1 (numerical taxonomy and multivariate analysis system) software package (Rohlf 2002). Cluster analysis was performed on the basis of genetic similarity matrix, and the resulting similarity co-efficients were used for constructing dendrogram using the unweighted pair group method with arithmetic average (UPGMA) with the SAHN module of NTSYS-pc (Sneath and Sokal 1973). The similarities between matrices based on different marker systems (RAPD and ISSR) were calculated using the standardized Mantel co-efficient (Mantel 1967).

## Results and discussion

Characterization of diversity has long been based mainly upon morphological traits. During the present investigation, morphological traits such as leaf color (green, dark green and yellow-green), stem (thickness and color), leaf size (large >70 mm^2^; medium 60–70 mm^2^; and small 50–60 mm^2^) and plant type (spreading or semi-erect) were used to identify *B. monnieri* accessions (Table 1). However, morphological variability is often restricted, characters may not be obvious at all stages of the plant development and appearance may be affected by the environment. Nowadays, a variety of different genetic markers has been proposed to assess genetic variability as a complementary strategy to more traditional approaches in genetic resources management. Molecular tools provide valuable data on diversity through their ability to detect variation at the DNA level. In this study, we have used 22 RAPD and 25 ISSR markers for identification of phylogenetic relationship among *B. monnieri* accessions.

### RAPD analysis

**Fig. 1:**
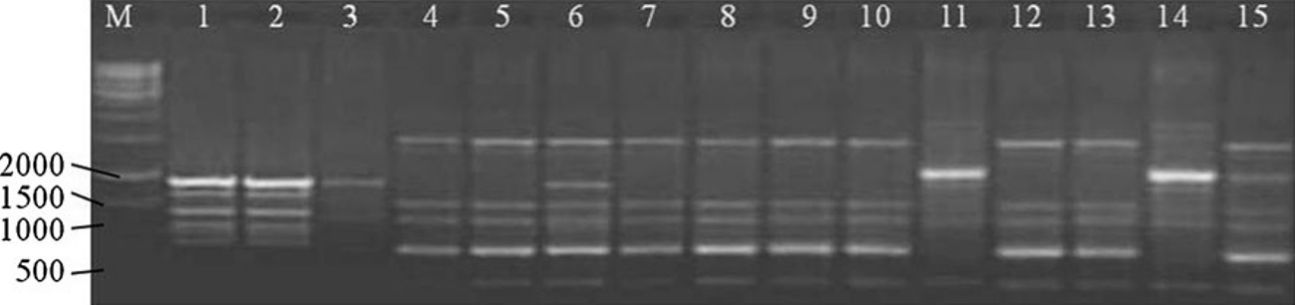
RAPD PCR product amplified by primer OPAI-06. *M* = 1 Kb DNA ladder, *lanes 1–15* represent *Bacopa monnieri* accessions described in Table 1

The RAPD analysis was carried out using decamer primers of different series of OPERON Technologies^®^ (OPA, OPAA, OPAB, OPAC, OPAD, OPAH, OPAI, OPB, OPBB and OPC) for DNA amplifications through PCR, out of which only 22 primers responded to all the accessions (Table 2). In RAPD profiling, a total of 197 reproducible bands were produced. The number of bands produced per primer ranged from 5 (OPAA-02) to 11 [OPC-05, OPAA-04, OPAB-07, OPAI-06 (Fig. 1) and OPAH-05]. Average number of bands per primer was 8.95, while average number of polymorphic bands was 8.50. The PIC value ranged from 0.363 (OPAI-03) to 0.908 (OPA-11) with an average 0.644. In all 22 RAPD primers were found to be polymorphic, out of which 16 primers were found to be 100 % polymorphic and remaining 6 exhibited variable percentage of polymorphism. Out of 197 bands obtained, 10 (5.07 %) were monomorphic and 187 (94.92 %) were polymorphic (Table 2). On the other hand, Darokar et al. (2001) reported only 48 % polymorphism with RAPD markers among geographically distinct accessions of *B. monnieri* and they concluded that the narrow genetic base in *B*. *monnieri* population could be attributed to the vegetative propagation of the species. This lack of RAPD variation may be possible due to highly heterozygous plants but that may not be uniform across the different populations throughout the country.

**Fig. 2:**
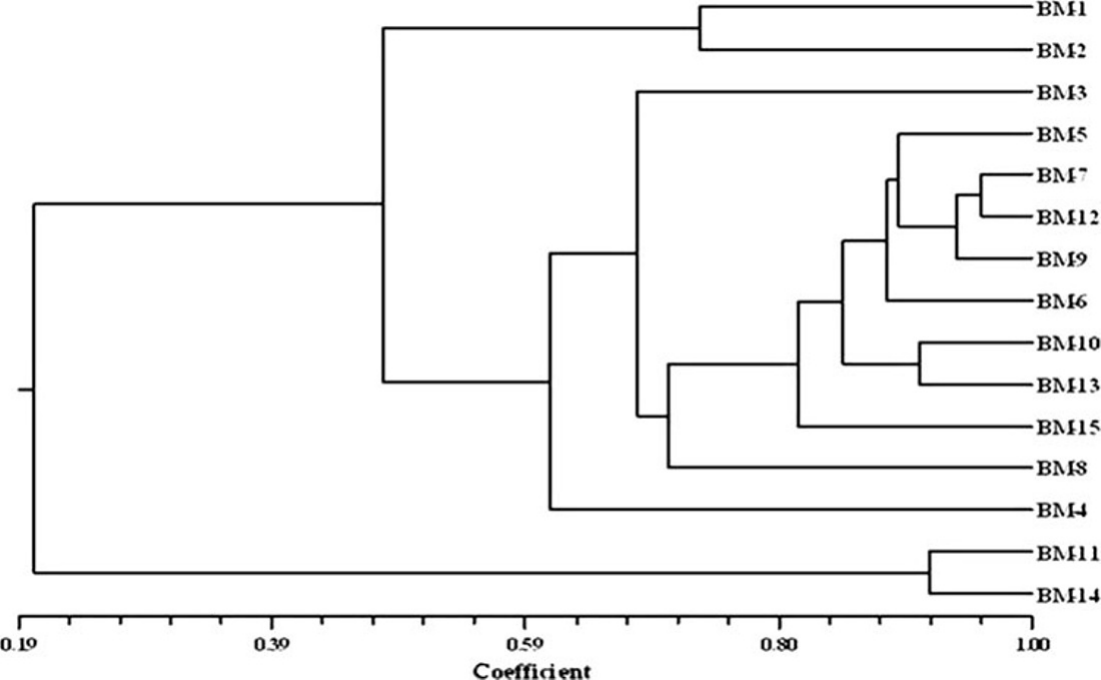
Dendrogram on the basis of the RAPD similarity matrix data by unweighted pair group method with average (UPGMA) cluster analysis

The genetic similarity co-efficients based on RAPD markers of the 15 studied Brahmi accessions illustrated that the highest similarity value (0.96) was recorded between BM-7 and BM-4, while the lowest similarity value (0.16) between BM-14 and BM-13 (data not shown). Cluster analysis was performed on the basis of similarity co-efficient generated from RAPD profiles. The cluster analysis divided all the Brahmi genotypes under study into two major groups (Fig. 2). Major group A was further divided into two sub groups. First subgroup comprising only two accessions BM-1 and BM-2 belonging to Hoshangabad and Dewas, respectively. Moreover, these two accessions were also similar in their morphological characteristics. Second subgroup held 11 accessions from diverse parts of Central India such as Khandwa (BM-3), Bhopal (BM-5), Rewa (BM-7), Singrauli (BM-12), Amarkantak (BM-9), Seoni (BM-6), Shivpuri (BM-10), Jabalpur (BM-13), Indore (BM-4 and BM-15) and Badwani (BM-8). In this subgroup, BM-3 and BM-4 showed further divergence from other accessions. Group B comprised only two accessions BM-11 (Satna) and BM-14 (JNKVV) and these accessions were quite diverged from others as this group was discretely placed at end of the cluster.

The genetic structure of plant populations reflects the interactions of many different processes such as the long-term evolutionary history of the species (e.g., shifts in distribution, habitat fragmentation, and/or population isolation), mutation, genetic drift, mating system, gene flow, and selection (Slatkin 1987; Schaal et al. 1998). All of these factors can lead to complex genetic structuring within populations. Genetic diversity is of great importance to the sustainability of plant populations (Wang et al. 2007). Effective conservation of a vulnerable species depends largely on the knowledge of patterns of genetic variation. For example, the spatial structure of genetic variation can provide information for sampling strategies for ex situ or in situ conservation (Torre et al. 2008).

Polymorphism in RAPD assay may arise due to deletion, addition or substitution of base within the priming site sequence (Williams et al. 1990). High diversity is the reflection of adaptation to environment, which is beneficial to its propagation, resources conservation, domestication of wild species and to screen specified locus. Geographically, isolated individuals tend to accumulate genetic variations during the course of environmental adaptations (Sarwat et al. 2008). This study is an attempt to establish the genetic diversity background in *B. monnieri* with RAPD markers. High levels of polymorphism found in the present investigation reveal that RAPD markers as a suitable tool for genetic diversity studies. This study will pave the way for detailed research to understand all the aspects of this divergence. RAPD has been successfully utilized for the identification of medicinal plants (Tochika-Komatsu et al. 2001; Um et al. 2001) and herbal medicinal components (Shinde et al. 2007). This technique has also been reported to be useful for the identification and genotyping of ornamental plants (De Benedetti et al. 2001) and other varieties of plant species (Temiesak et al. 1993).

### ISSR analysis

**Fig. 3:**
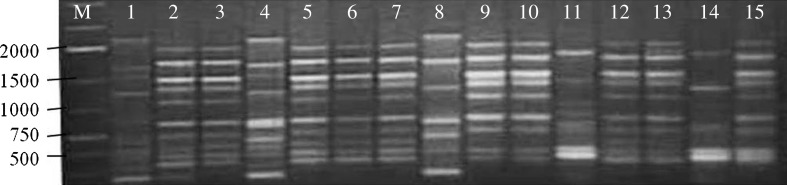
ISSR PCR product amplified by primer UBC-876. *M* = 1 Kb DNA ladder, *lanes 1–15* represent *Bacopa monnieri* accessions described in Table 1

The ISSR analysis was carried out using a set of 100 UBC primers for DNA amplifications through PCR, out of which only 25 primers responded to all the accessions (Table 2). In ISSR profiling, a total of 284 bands were produced. The number of bands produced per primer ranged from 6 (UBC 812) to 16 (UBC 811, UBC 842). ISSR amplification pattern amplified by primer UBC 876 illustrated in Fig. 3. Average number of bands per primer was 11.36, while average number of polymorphic bands was 10.8. All the primers were found to be polymorphic, out of 25 ISSR primers, the lowest polymorphism 80 % was shown by primer UBC 808, and 15 primers were 100 % polymorphic. The range of PIC value of the primers was 0.418 (UBC 895) to 0.836 (UBC823) with an average 0.664. Out of 284 bands, 14 (4.93 %) were monomorphic and 270 (95.07 %) were polymorphic with the average of 95.22 polymorphic bands per primer (Table 2). Monomorphic bands are those which are present in all individuals, polymorphic are present in one or more but not all individuals and unique ones are present in at least one individual not in any other (Mehetre et al. 2004).

**Fig. 4:**
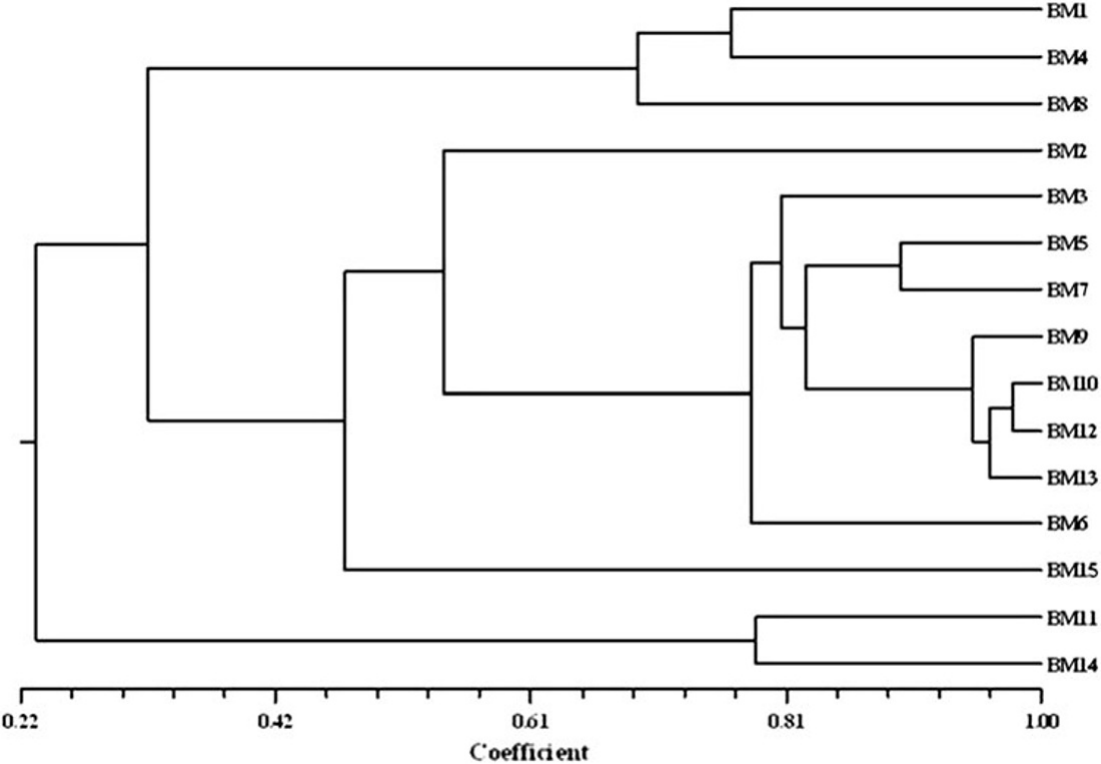
Dendrogram on the basis of ISSR similarity matrix data by unweighted pair group method with average (UPGMA) cluster analysis

Cluster analysis was performed on the basis of similarity co-efficient generated from ISSR profiles. The cluster analysis grouped all the Brahmi genotypes under study in two groups (Fig. 4). Major group A was divided into two sub groups, first subgroup containing only three accessions, namely, BM-1 (Hoshangabad), BM-4 (Indore) and BM-8 (Badwani). Second subgroup contains ten accessions as BM-2 (Dewas), BM-3 (Khandwa), BM-5 (Bhopal), BM-7 (Rewa), BM-12 (Singrauli), BM-9 (Amarkantak), BM-6 (Seoni), BM-10 (Shivpuri), BM-13 (TFRI-Jabalpur), and BM-15 (Indore). Group B containing only two accessions, namely, BM-11 (Satna) and BM-14 (JNKVV). These accessions are diverse from other accessions and placed at end of the cluster.

The ISSR technology is sensitive to considerable levels of genetic variation, providing a very useful molecular tool for studying population genetics on a wide range of plant species, as well as for identifying species, cultivars, or population of the same species (Zietkiewicz et al. 1994; Raina et al. 2001; Wang et al. 2009). ISSR analysis is a PCR-based method with advantages of low-cost and high-efficiency as compared with other DNA genotyping techniques. The results indicated that ISSR markers have been successfully utilized for assessing the genetic diversity and revealed a remarkable molecular discrimination between the 15 Brahmi accessions under study. Moreover, (Galvan et al. 2003) concluded that ISSR would be a better tool than RAPD for phylogenetic studies. Nagaoka and Ogihara (1997) have also reported that the ISSR primers produced several times more information than RAPD markers in wheat. The number of potential ISSR markers depends on the frequency of microsatellites, which changes with species (Depeiger et al. 1995), So that the potential for integrating ISSR–PCR into plant improvement program is enormous and their applications in different crop species are sufficiently reviewed (Reddy et al. 2002).

### RAPD–ISSR combined analysis

RAPD and ISSR techniques have been widely employed in the assessment of genetic relationships both within and between plant species (Joshi et al. 2000; Patzak 2001; Raina et al. 2001). RAPD and ISSR are simple, provide a quick screen for DNA polymorphism and very small amounts of DNA are required. In addition, information on template DNA sequence is not needed (Jasieniuk and Maxwell 2001). During the present work, a combination of RAPD and ISSR markers has been used to determine the genetic affinities between Brahmi accessions at the DNA level. The results indicated close correspondence between the similarity matrices of both RAPD and ISSR individually or combined, hence both the marker systems can be used effectively in determining genetic relationship of Brahmi accessions. The genetic variation through RAPD and ISSR markers has been highlighted in a number of medicinal plants (Bai et al*.*^1997^; Rout et al. 1998; Pal and Raychaudhuri 2003; Rout 2006). The results show that both the marker systems are efficient to distinguish 15 accessions of Brahmi and to reveal molecular relationship among them.

**Fig. 5:**
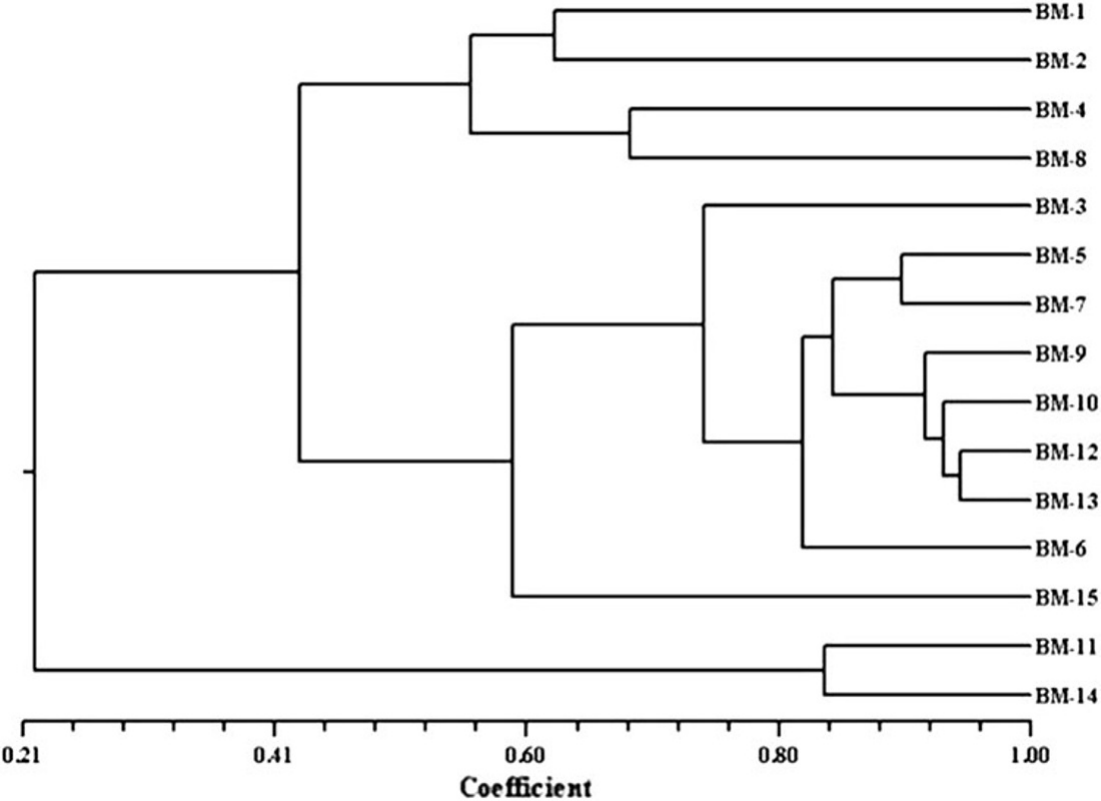
Dendrogram on the basis of combined (RAPD + ISSR) similarity matrix data by unweighted pair group with average (UPGMA) cluster analysis

The similarity co-efficients of the 15 Brahmi accessions based on RAPD and ISSR markers ranged from 0.945 to 0.179 among all the genotypes. Accessions BM-13 and BM-12 showed the highest similarity index (0.945), while the lowest (0.179) between BM-14 and BM-7 (Table 3). The matrices were compared using Mantel (1967) test and revealed high correlation values between the two markers (*r* = 0.71384, *p* > 0.001), which indicates a good fit among RAPD and ISSR marker systems. Cluster analysis performed from combining data of both markers generated a dendrogram separating the genotypes into two clusters. Cluster I further divided into two sub clusters. First sub cluster contained four Brahmi accessions, namely, BM-1, BM-2, BM-4 and BM-8. Within the second sub cluster comprising nine accessions (BM-3, BM-5, BM-7, BM-9, BM-10, BM-12, BM-13, BM-6 and BM-15), BM-12 was very closely related to BM-13 (0.945) followed by BM-9 (0.941). Cluster II comprised only two accessions BM-11 and BM-14 with a genetic similarity index of 0.838. The dendrograms generated by both approaches individually (RAPD and ISSR) were in strong agreement with each other as well as with combined (RAPD-ISSR) approach. Two major groups were obtained and most of the related accessions were found to be grouped together (Fig. 5). Similarly, Mathur et al. (2003) found two clusters (one major and one minor) during their study on *B. monnieri* accessions collected from different geographical regions of India using various morphological characteristics. In our study, accessions BM-11 and BM-14 were always grouped together using either approach. The morphological analysis of these accessions (BM-11 and BM-14) also supports the similar clustering patterns as both spreading plant type have medium leaf size, dark green leaf color with thin green stem.

**Table 3:** Jaccard’s similarity co-efficient among *Bacopa monnieri* accessions using RAPD and ISSR combined data

	BM-1	BM-2	BM-3	BM-4	BM-5	BM-6	BM-7	BM-8	BM-9	BM-10	BM-11	BM-12	BM-13	BM-14	BM-15
BM-1	1.000														
BM-2	0.627	1.000													
BM-3	0.295	0.527	1.000												
BM-4	0.636	0.548	0.360	1.000											
BM-5	0.310	0.518	0.766	0.404	1.000										
BM-6	0.412	0.634	0.689	0.481	0.831	1.000									
BM-7	0.339	0.552	0.737	0.429	0.898	0.850	1.000								
BM-8	0.559	0.498	0.383	0.685	0.421	0.497	0.451	1.000							
BM-9	0.315	0.521	0.767	0.397	0.874	0.807	0.866	0.456	1.000						
BM-10	0.309	0.526	0.730	0.405	0.842	0.805	0.820	0.434	0.921	1.000					
BM-11	0.282	0.251	0.198	0.273	0.200	0.215	0.190	0.250	0.204	0.200	1.000				
BM-12	0.315	0.526	0.767	0.401	0.867	0.833	0.851	0.438	0.941	0.929	0.201	1.000			
BM-13	0.298	0.504	0.767	0.382	0.827	0.797	0.805	0.423	0.889	0.933	0.189	0.945	1.000		
BM-14	0.255	0.224	0.183	0.252	0.187	0.199	0.179	0.232	0.190	0.192	0.838	0.190	0.182	1.000	
BM-15	0.354	0.444	0.528	0.436	0.571	0.601	0.584	0.454	0.609	0.598	0.294	0.636	0.622	0.275	1.000

**Fig. 6:**
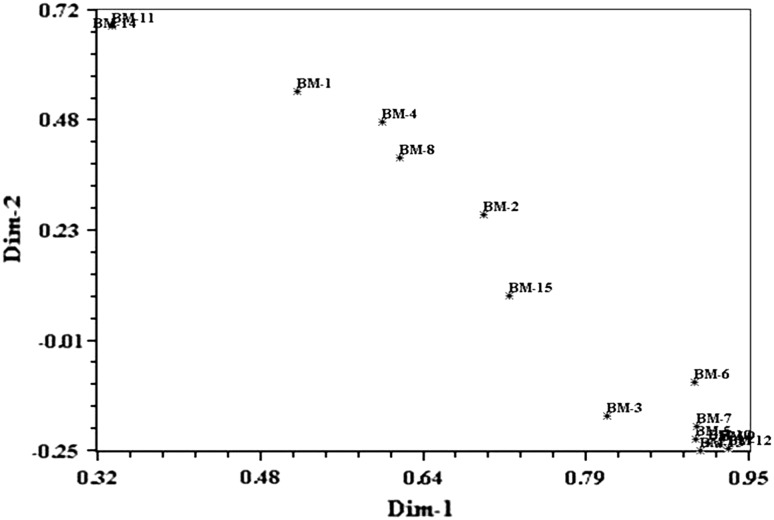
Two dimensional plot of 15 Brahmi accessions PCA (principal component analysis) based on combined (RAPD + ISSR) UPGMA (Table 1 for accessions label)

The cluster analysis of 15 accessions of *B. monnieri* with molecular approaches revealed that accessions collected from nearby locations fell into different clusters and those from geographically different locations fell in the same cluster. Occasionally, some of the accessions from nearby geographical locations fell in the same cluster. These results imply that multiplicity of factors including the geographical locations were responsible for the selection of genotypes that got naturalized at the sites of collections. Similar results have been reported earlier for several plant species (Gupta et al. 1991; Dias et al. 1993; Amurrio et al. 1995; Rabbani et al. 1998). It was interesting to note that accessions BM-11 and BM-14 from relatively nearer geographical origins with distinct phenotypes from other accessions (Table 1) formed a distinct cluster on the basis of dendrogram (Figs. 2, 4) and two dimensional PCA analyses (Fig. 6).

It was also observed from this study that some accessions were collected from the similar climatic zone does not exhibit similar morphological characteristics. This suggests that morphologic divergence observed cannot solely be attributed to environmental variations but possibly to a genetic restructuring evolved in the populations. However, to test this hypothesis, it would be necessary to collect extensive morphologic data and subject them in the evaluation of the distribution of variability.

In general, the molecular classifications more or less follow morphological classification. However, in our study, discrepancy exists between morphological characteristics and molecular data. This might be most likely the result of the primary stages of Brahmi breeding, during which most cultivars and varieties derived from open pollination, artificial selection, and are maintained by vegetative propagation. In similar studies in olive (Hagidimitriou et al. 2005) and almond cultivars (MirAli and Nabulsi 2003), very low correlation was found between the molecular and morphological data. Based on the result, Hagidimitriou et al. (2005) proposed that morphological traits were not reliable in estimating genetic relationships among diverse groups of cultivars.

## Conclusions

It can be concluded that RAPD and ISSR markers may be a more useful tool for the identification of Brahmi than morphological characters. The present findings can help the genetic variation analysis among different accessions of Brahmi. On the basis of the findings of the present study, we can conclude that Central India is a good source of genetic variability. However, detailed study is desirable to understand all the aspect related to variations. Hence, further information will be necessary on patterns of gene flow within and between population and its effects on reproductive and demographic processes, to assess its impact on population viability. The accessions showed a considerable level of genetic diversity, indicating a high genetic variability in the population. The genetic variability in a gene pool is normally considered as being the major resource available for breeding programs.

## Abbreviations

SSR: Simple sequence repeat
AFLP: Amplified fragment length polymorphism
RAPD: Random amplified polymorphic DNA
ISSR: Inter simple sequence repeats
RFLP: Restriction fragment length polymorphism
PIC: Polymorphic information content
DNA: Deoxyribonucleic acid
PCR: Polymerase chain reaction
UPGMA: Unweighted pair group method with the arithmetic averaging algorithm
PCA: Principal component analysis
